# The London Hospital's Appeal

**Published:** 1920-06-05

**Authors:** 


### THE LONDON HOSPITAL'S APPEAL.

There is not a man in the hospital world who will not sympathise with the appeal issued at the eleventh hour by Lord Knutsford on May 28. Among the many hard cases in London at the present time—the provincial hospitals are more fortunately situated—not even St. Bartholomew's Hospital has been harder hit by want of money. The problem is to raise at least £100,000 for the London Hospital without impeding the appeals of St. Bartholomew's and the sister hospitals. Like the chairmen of the latter, Lord Knutsford has held his hand as long as he possibly could, and this should be gratefully remembered. For, it must not be forgotten, the law of self-preservation under the voluntary system can be put into execution disinterestedly; since if the London Hospital, which is the biggest institution in the Metropolis, does not triumph over its difficulties, some other hospital in London may feel obliged to capitulate. That capitulation would be infectious; but so will be the success of the London Hospital's appeal.

What has been the immediate response? The first week will have given some indication of the chances of ultimate success. The immediate result of the appeal was encouraging. The post on Saturday morning, May 29, produced nearly £3,000, and, in spite of inaccurate reports in the preceding evening's papers, £400 arrived on the first day. The money, it is interesting to observe, first came chiefly in small sums and from people in or near Whitechapel, who have had the benefit of observing the placards in the front of the hospital. One gentleman telegraphed his desire to know the cost of the hospital's bills for one week, which has been followed by his undertaking to pay such cost, viz., £4,000. The example thus set may, we hope, prove contagious. We are informed that an anonymous donor has sent another £4,000 for this purpose, and as we go to press the result to date of the London's appeal is £20,000. Without exaggeration, therefore, it may be fairly said that Mr. E. W. Morris and his committee are justified in feeling that State hospitals are a long way off. The suggestion is that, if necessary, the Treasury may undertake some specific item in the bill—for instance, the cost of the institutional treatment of insured persons.

But, as we said at the beginning, this appeal is of general importance, and in the long run the other London hospitals cannot fail to benefit from its success. Let us note a few points of general application. While each London hospital appeals to the present amorphous mass of Londoners, each has at least some local support and its own way of inviting it. The small sums from which the first fruits of the London Hospital Appeal were garnered suggest, as The Hospital has urged of late, that organisation and directness must be greatly improved. The Metropolitan Hospital is trying to improve its own position in this regard, and the other hospitals must do the same after their own tradition and character. There could be no greater mistake than to fancy that the methods in favour at the London Hospital are, or are intended to be, of universal application. Servile copying is worse than useless. Given the same conviction and initiative, carefully applied to local differences, other methods will be equally successful elsewhere. Incidentally we wonder, as we write in advance, if the Red Cross or other general fund has attempted to make any use of the crowd, so free with its money, on Epsom Downs for the Derby celebration. But though these general appeals must not be neglected they are the province of central funds, not of individual hospitals. It would be a mistake for the other London hospitals to be timid because, at the eleventh hour, the London has placed its trump card upon the table. St. Bartholomew's has already raised the sum of £130,000. The number of individual recorded contributors, so far, to the appeal exceeds 12,000, which is claimed to be the largest ever obtained for a Hospital appeal. St. Bartholomew's, therefore, deserves well by reason of the energy already displayed. It is the less large hospitals in London whose position claims immediate consideration. Their course is plain. They have to decide whether to rely *mainly*, for they must rely to a large extent, upon separate appeals; or whether *to combine with these* local efforts a joint collective campaign. The immediate point is not to neglect the former. A collective appeal can be delayed: collective action is slow action; local appeals in local papers, cinematographs, and so forth, cannot, and should not, wait. If these means are despised it will be because they are not understood, not because they are not valuable. What we really need to breast the hill in London are a little cheerfulness, conviction, and common sense. If the London Hospital appeals over the heads of the smaller hospitals, there is plenty of money at the latter's feet which method and organisation can gather.

